# Severe Metformin Poisoning Successfully Treated with Simultaneous Venovenous Hemofiltration and Prolonged Intermittent Hemodialysis

**DOI:** 10.1155/2018/3868051

**Published:** 2018-05-08

**Authors:** Dovile Leonaviciute, Bo Madsen, Anne Schmedes, Niels H. Buus, Bodil S. Rasmussen

**Affiliations:** ^1^Department of Anaesthesiology and Intensive Care Medicine, Aalborg University Hospital, Aalborg, Denmark; ^2^Department of Nephrology, Aalborg University Hospital, Aalborg, Denmark; ^3^Department of Biochemistry and Immunology, Lillebaelt Hospital, Vejle, Denmark; ^4^Department of Clinical Medicine, Aalborg University, Aalborg, Denmark

## Abstract

Metformin poisoning is a life-threatening condition with a high mortality rate. We present a patient case of metformin poisoning following intake of 80 g metformin resulting in severe lactate acidosis with a nadir pH of 6.73 and circulatory collapse, successfully treated with addition of prolonged intermittent hemodialysis (HD) to continuous venovenous hemofiltration (CVVH). The patient's pH became normal 48 hours after metformin ingestion during simultaneous CVVH and addition of 22 hours of intermittent HD in the ICU. The highest metformin level was found to be 991 *μ*mol/L (therapeutic range 3.9–23.2 *μ*mol/L). We conclude that in cases of severe metformin poisoning with circulatory shock and extreme lactic acidosis, the usual CVVH modality might not efficiently clear metformin. Therefore, additional prolonged HD should be considered even in the state of cardiovascular collapse with vasopressor requirement.

## 1. Introduction

Metformin is widely used and is the most frequently prescribed oral antidiabetic drug of the biguanide family [1]. Metformin inhibits hepatic gluconeogenesis and glycogenolysis and enhances peripheral glucose utilisation in patients with non-insulin-dependent diabetes [2, 3]. Metformin use is generally safe and well tolerated. However, lactic acidosis is a well-known complication to metformin treatment, especially in intentional overdose or in cases of renal insufficiency. Metformin poisoning is a life-threatening condition with a very high mortality rate. With the modern intensive care treatment, mortality ranges from 50% to 30% [4]. However, those intentional overdose patients with severe acidosis (pH less than 6.9) have a mortality rate as high as 83% [5]. We present a patient case of metformin poisoning following intake of 80 g metformin resulting in severe lactate acidosis with a nadir pH of 6.73 and circulatory collapse, successfully treated with addition of prolonged intermittent hemodialysis (HD) to continuous venovenous hemofiltration (CVVH).

## 2. Case Presentation

A 54-year-old patient with a body weight of 66 kg and a medical history of anxiety, type 2 diabetes, and previous stroke was admitted to the local hospital one hour after ingestion of 80 grams of metformin in a suicide attempt. At the Emergency Department, an aspiration of the gastric content was performed followed by administration of activated charcoal. The patient developed vomiting and diarrhoea. Arterial blood gas showed pH 7.33, PaCO_2_ 4.8 kPa, PaO_2_ 12.8 kPa, bicarbonate 19.3 mmol/L, base deficit 7 mmol/L, lactate 5.9 mmol/L, and blood glucose 17.9 mmol/L. The patient was transferred to a university hospital. Three hours after ingestion of metformin, the metabolic acidosis had deteriorated with serum lactate 8.5 mmol/L and pH 7.23. Urgent hemodialysis (HD) was performed for 4 hours (h) by the nephrology department with a blood flow of 150 mL/min and a Fresenius FX60 dialyzer (Fresenius, Bad Homburg, Germany) with a surface area of 1.4 m^2^. During HD, the patient became increasingly acidotic and circulatory unstable and was admitted to the intensive care unit (ICU) for circulatory support and continuous CVVH. CVVH (Aquarius, Edwards Lifesciences, Irvine, USA) was initiated with a blood flow of 300 mL/min and a replacement fluid rate of 2000 mL/h (30 mL/kg/h) with 35 mmol/L bicarbonate in Accusol 35 (Baxter Healthcare Ltd., Norfolk, United Kingdom) with 50% of the replacement fluid infused prefilter and 50% postfilter via a dialysis catheter placed in the right internal jugular vein. Dialyzer used was Aquamax HF 12 (Nikkiso, Bellco, Italy) with a surface area of 1,2 m^2^. Because of filter clotting, CVVH stopped after one hour. The metabolic acidosis escalated further with a pH decreasing to 6.73 and a serum lactate above the upper measuring limit of 30 mmol/L (ABL800 Flex, Radiometer, Copenhagen, Denmark). The patient was less conscious but managed to hyperventilate to a PaCO_2_ of 2.6 kPa and base deficit of 29 mmol/L. He developed severe hypotension, oliguria, hypothermia with core temperature 32.2°C, and hypoglycaemia with a nadir blood glucose of 2.3 mmol/L. Noradrenaline infusion was initiated with increasing dosage to keep mean arterial pressure above 60 mmHg. Bicarbonate 8.4% infusion was continued together with volume substitution with crystalloids. CVVH was restarted after 3 hours. Active external rewarming with forced-air warming blanket (Model 30000, 3M Health Care, St. Paul, USA) and maximal possible temperature of 39°C on CVVH was used to treat the hypothermia, but the temperature did not increase. As the patient's condition deteriorated, the hemodynamic instability progressed, cardiac arrhythmias occurred, and serum lactate remained immeasurably high. Focus Assessed Transthoracic Echocardiography (FATE) revealed a normal systolic function of both ventricles.

**Figure 1:**
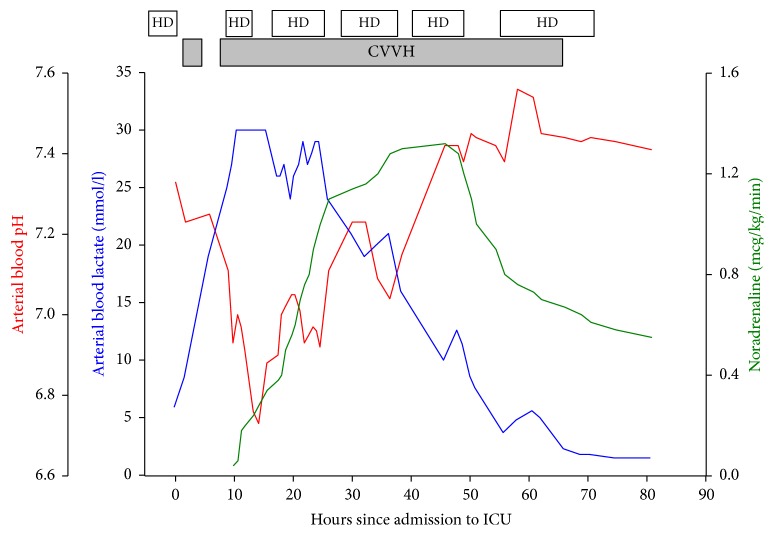
Measurements of arterial blood pH (red) and lactate (blue) together with the infusion rate of noradrenaline (green) in the hours following admission to the intensive care unit (ICU). HD denotes hemodialysis and CVVH continuous venovenous hemofiltration.

Because CVVH alone failed to stabilise the patient's condition and despite of hemodynamic instability and vasopressor requirement (noradrenaline infusion 0.4 mcg/kg/min), a decision was made to start HD simultaneously with CVVH. An extra dialysis catheter was placed in the left internal jugular vein and intermittent HD with a blood flow of 300 mL/min, Fresenius FX80 dialyzer (Fresenius, Bad Homburg, Germany) with a surface area of 1.8 m^2^, with the highest possible concentration of bicarbonate buffer of 40 mmol/L on HD machine, was simultaneously performed for 4 h and repeated for 8 h on the first day. On the second day, HD was performed twice for 8 h and on the third day for 16.5 h, while CVVH was continued simultaneously for almost 58 h. Progression of the acidosis occurred with a drop in pH whenever HD was paused (Figure 1).

On the second day in the ICU, the patient was intubated and mechanical ventilation was initiated, because of respiratory insufficiency due to increasing PaCO_2_. Only 48 h after metformin ingestion and 36 h in the ICU with cardiopulmonary support using very high doses of noradrenaline infusion (up to 1.4 mcg/kg/min) and CVVH combined with 22 h in total of intermittent HD with bicarbonate buffer, the patient's pH finally normalized. Serum lactate was still elevated but decreased very slowly to 2 mmol/L on the third day during the continued treatment with CVVH and intermittent HD. After 58 h on both CVVH and intermittent HD, the CVVH was discontinued and the patient remained on intermittent HD because of acute oliguric renal failure until day 15. The patient had an increase of international normalized ratio (INR) from 1.2 at the day of admission to a maximal value of 2.0 at day 2. The patient's condition improved gradually and he was discharged from the hospital after 7 weeks. At that time, the patient was still nourished via a percutaneous endoscopic gastrostomy tube due to dysphagia and used a walker due to polyneuropathy. The patient's renal function on discharge was normal.

A metformin plasma concentration, determined by high pressure liquid chromatography tandem mass spectrometry (Waters Acquity UPLC, Xevo TQ-S), was later measured from the plasma obtained 14 h after poisoning (after 4 h of HD and one hour of CVVH) and was found to be as high as 991 *μ*mol/L (therapeutic range 3.9–23.2 *μ*mol/L). 48 h after poisoning, remaining on CVVH and additional 20 hours of intermittent HD, metformin concentration was reduced to 327 *μ*mol/L. After additional 8 hours of HD and CVVH, metformin concentration was further reduced to 84.4 *μ*mol/L at 62 h after poisoning.

## 3. Discussion

Severe metformin poisoning often presents with a profound lactic acidosis followed by collapse of the cardiovascular system. Symptoms of metformin poisoning are diffuse with abdominal pain, nausea, vomiting, hypothermia, decreased level of consciousness, and circulatory instability leading to multiorgan failure. The circulatory instability is due to peripheral vasoplegia as described in many case reports where low systemic vascular resistance was measured [6]. We observed a clinical picture of a hyperdynamic circulatory state where FATE revealed normal contractility of the heart together with severe systemic hypotension. Vasoplegia and vasodilatation can explain hypothermia, which is a common symptom of metformin poisoning [6].

Treatment of metformin poisoning is symptomatic and supportive and there is no antidote available. Typical treatment strategies consist of correcting acidosis with intravenous sodium bicarbonate and decreasing the blood levels of metformin. Further prevention of gastrointestinal absorption can be achieved with activated charcoal. In case of renal insufficiency, renal replacement therapy is the only option for metformin removal and acidosis correction.

Metformin is readily dialyzable. It is a small molecule with a molecular weight of 165 Da, not protein bound, and after gastrointestinal absorption it rapidly moves into the tissue compartment and has a large volume of distribution of 63–276 L (1–5 L/kg) [1]. Lalau et al. previously demonstrated a biphasic pattern of metformin elimination according to a two-compartment model. This two-compartment model suggests that a brief hemodialysis session is not sufficient in eliminating metformin due to a rebound phenomenon [7]. These pharmacokinetic properties indicate a need of prolonged dialysis for metformin elimination. Several case reports describe favourable outcome after severe metformin poisoning treated with prolonged intermittent HD. These reports suggest that prolonged HD (>15 h) is needed and indicated in patients, who suffer from a severe overdose and who are able to tolerate HD [2, 8]. In our case, 62 h after ingestion and with ongoing treatment with CVVH and additional 32 h of intermittent HD, the metformin level was still elevated to 84.4 *μ*mol/L, emphasizing the need for prolonged HD in severe metformin overdose.

Usually intermittent HD is poorly tolerated in critically ill patients with hemodynamic instability [9]. In the ICU setting CVVH or continuous venovenous hemodiafiltration (CVVHDF) is used because fluid shifts are less profound and therefore applicable in hemodynamic unstable patients. However, these modalities need high ultrafiltration rates for successful treatment in case of metformin intoxication [10], but clearance of metformin by continuous venovenous treatment modalities is still 3-4 times less than clearance by conventional HD [1, 11]. Panzer reported successful use of two CVVH machines simultaneously for hemodynamically unstable patient to facilitate high volume CVVH [12]. Friesecke has successfully applied the same approach with two dialysis catheters and used discontinuous hemofiltration (highflux) in addition to CVVH for severe circulatory unstable patient. To our knowledge, the use of intermittent HD in a patient with metformin poisoning with severe hemodynamic instability and such dependence on vasopressors is described only once [13]. However, the placement of two dialysis catheters in the jugular vein position may theoretically lead to a recirculation between them, if the tips of the catheters are placed close to each other. The jugular and femoral placement of the catheters will eliminate this possibility.

Substitution with bicarbonate in case of lactic acidosis is controversial as there are concerns that treatment with bicarbonate can increase intracellular acidosis [14]. However, bicarbonate treatment is widely used to stabilise metabolic acidosis in cases of metformin poisoning as described in the literature. We used high doses of bicarbonate as intravenous infusion and highest possible concentration of bicarbonate buffer in the HD machine simultaneously with CVVH with 35 mmol/L bicarbonate in the Accusol 35 (Baxter) replacement fluid. We observed worsening of the acidosis and cardiovascular status when HD was paused and we presume this deterioration to be due to lack of bicarbonate buffer. Renal replacement therapy, including conventional HD and CVVH, offers both theoretical and practical advantages over bicarbonate infusion. These allow for isovolemic correction of the metabolic acidosis while removing metformin and lactate [6].

Recent recommendations for metformin poisoning from the Extracorporeal Treatments in Poisoning Workgroup advocate for intermittent HD, but continuous renal replacement therapies may be considered if HD is unavailable [1]. Initiation of dialysis is suggested in very severe cases, when lactate concentration is >15 mmol/L and pH < 7.0 together with shock or organ failure [1]. In our opinion, these recommendations are more applicable in situations of metformin-associated lactic acidosis, as in acute high dose metformin poisoning the acidosis accelerates very fast and the patient's condition deteriorates dramatically with development of circulatory collapse.

We therefore suggest that in cases of acute high dose metformin poisoning HD should be initiated earlier than that stated in the recommendations.

## 4. Conclusion

In cases of severe metformin poisoning with shock and extreme lactic acidosis usual CVVH modality might not effectively clear metformin and additional prolonged intermittent HD should be considered instead or together with CVVH, even in the state of cardiovascular collapse with vasopressor requirement.
